# Pediatric Enterovirus Encephalitis: Clinical, Neuroimaging, and Prognostic Insights From a Single-Center Study in the UAE

**DOI:** 10.7759/cureus.109197

**Published:** 2026-05-19

**Authors:** Mohamed E Elkafafi, Ahmed Ghorab, Yaser Ali, Rajesh Phatak

**Affiliations:** 1 Pediatric Intensive Care, Burjeel Hospital, Abu Dhabi, ARE; 2 Medicine, East Lancashire NHS Trust, Manchester, GBR; 3 Pediatrics, Burjeel Hospital, Abu Dhabi, ARE

**Keywords:** acute leukoencephalopathy with restricted diffusion (alerd), central nervous system infections, cerebrospinal fluid, encephalitis, middle east, neuroimaging, neurologic manifestations, pediatrics, polymerase chain reaction, supportive care

## Abstract

Enteroviral encephalitis in children is rare but can lead to severe neurological complications. This study describes the clinical, neuroimaging, and laboratory features of pediatric enteroviral encephalitis at Burjeel Hospital, Abu Dhabi, and explores, in an exploratory and hypothesis-generating manner, associations between clinical findings, laboratory results, neuroimaging, and patient outcomes. This is a small descriptive case series (n = 9), and all findings should be interpreted accordingly.

A retrospective study included nine pediatric patients with enteroviral encephalitis confirmed by CSF PCR; other etiologies were excluded per predefined inclusion and exclusion criteria. The median age was five years, with a male predominance (n = 7; 77.8%). Symptoms included fever (9/9; 100%), vomiting (8/9; 88.9%), headache (7/9; 77.8%), and neck stiffness (7/9; 77.8%). Seizures occurred in one patient (1/9; 11.1%). Total hospital stays ranged from 2 to 15 days (median three days); PICU stays ranged from 1 to 12 days (median one day).

As an exploratory observation, neuroimaging abnormalities showed an association with prolonged PICU stays (rs = 0.694, p = 0.038) and adverse outcomes, while CRP levels did not (rs =− 0.548, p = 0.127); these are hypothesis-generating findings only and not confirmatory. One patient (1/9; 11.1%) with ALERD on MRI had the most severe outcome, including long-term neurological sequelae. Eight of nine patients (8/9; 88.9%) achieved full neurological recovery with supportive care alone. This exploratory case series suggests a potential role for early neuroimaging in clinical decision-making and contributes region-specific data from the UAE. Prospective multicenter studies are required to validate these observations.

## Introduction

Enteroviruses are among the most common disease-causing viruses worldwide, responsible for a wide spectrum of illnesses ranging from mild respiratory infections to severe neurological disease, including encephalitis [1]. The class encompasses enteroviruses, coxsackieviruses, echoviruses, polioviruses, and rhinoviruses.

Encephalitis in children carries an estimated incidence of 16/100,000 worldwide, affecting boys and girls equally [2]. The most common cause globally is viral, with herpes simplex virus (HSV) predominating. Other recognized viral agents include varicella-zoster virus (VZV), cytomegalovirus (CMV), Epstein-Barr virus (EBV), adenovirus, and enterovirus. Arthropod-borne viruses, including West Nile virus (WNV) and Japanese encephalitis virus (JEV), are also well-recognized etiologies. Bacterial causes include *Mycobacterium tuberculosis*, *Listeria monocytogenes*, *Mycoplasma pneumoniae*, *Borrelia*, and *Rickettsia* [2]. In immunocompromised individuals, fungal and parasitic etiologies must also be considered. Non-infectious immune-mediated causes, including ADEM, anti-NMDAR encephalitis, and anti-MOG encephalitis, form an increasingly recognized category [3]. On occasion, no etiology is identified despite thorough investigation.

Encephalitis in children requires careful management, ideally in a PICU setting. Empirical IV acyclovir should be initiated pending HSV exclusion, with subsequent management directed by etiology. For most viral encephalitides, care is supportive. Known acute complications include seizures, reduced consciousness, cerebral edema, acute leukoencephalopathy with restricted diffusion (ALERD), and syndrome of inappropriate antidiuretic hormone secretion (SIADH) [4]. Long-term neurodevelopmental sequelae affect nearly half of childhood encephalitis survivors, including cognitive impairment, motor dysfunction, epilepsy, and developmental delay [5].

Although enteroviral encephalitis is a recognized cause of pediatric CNS disease, data from the Middle East remain scarce, particularly regarding clinical outcomes, neuroimaging features, and prognostic factors. Regional differences in enterovirus serotypes, environmental factors, and healthcare access may modulate disease expression, underscoring the need for region-specific data.

This study had two aims: (1) to describe the clinical, laboratory, and neuroimaging features of nine pediatric patients with PCR-confirmed enteroviral encephalitis managed at Burjeel Hospital, Abu Dhabi; and (2) to explore, in an exploratory and hypothesis-generating manner, potential associations between these features and patient outcomes.

## Materials and methods

This was a retrospective observational study conducted at Burjeel Hospital, Abu Dhabi, UAE, from January 2023 to December 2023 (12 months). All eligible patients meeting the inclusion criteria during this period were included consecutively; no eligible patients were excluded. Data were extracted from hospital electronic medical records.

Ethical approval

This study received a formal waiver by the Burjeel Medical City Research Ethics Committee (as applicable under institutional policy for retrospective, anonymized case series). Written informed consent for the use of anonymized clinical data was obtained from the guardians of all patients prior to data collection.

Enteroviral encephalitis was defined as acute neurological dysfunction (altered consciousness, seizures, or focal neurological signs) with CSF PCR confirmed positive for enterovirus, consistent with the International Encephalitis Consortium (IEC) 2013 criteria [6]. Alternative diagnoses were excluded by negative CSF PCR for HSV, VZV, and other relevant pathogens and by clinical assessment. We acknowledge that some cases may represent predominantly meningitic rather than true encephalitic involvement, given the mild clinical course observed in the majority.

Inclusion criteria

Pediatric patients aged 0-18 years with positive CSF PCR for enterovirus and clinical features consistent with CNS infection. 

Exclusion criteria

All neuroimaging (CT or MRI) was performed on clinical indication and reported by consultant pediatric radiologists at our institution. CSF PCR was performed using a validated pan-enteroviral PCR platform in our accredited laboratory; enterovirus serotyping was not available. Most patients were male (7/9; 77.8%). All patients were admitted to the PICU at Burjeel Hospital.

Data collected included demographics, clinical symptoms, laboratory data (CSF analysis, full blood count, CRP, procalcitonin [PCT]), neuroimaging findings (CT/MRI), management details, and patient outcomes. “Full recovery” was defined as discharge with no objective neurological deficit on clinical examination. “Persistent sequelae” was defined as the presence of one or more neurological deficits on examination at the time of discharge.

Descriptive statistics (median, range, N/%) were used to summarize the data. Spearman's rank correlation analyses were performed to explore associations between clinical, laboratory, and radiological findings. These analyses are strictly exploratory and hypothesis-generating only and should not be interpreted as confirmatory evidence given the sample size, the absence of multiple comparisons correction, and the inherent risk of type I error in small cohorts. A p-value of < 0.05 was used solely as the threshold for exploratory significance.

## Results

Summary

Nine patients aged 6 weeks to 10 years (median age 5 years; 7/9 male, 77.8%) were included. All nine (9/9; 100%) presented with fever. Other presenting symptoms included vomiting (8/9; 88.9%), headache (7/9; 77.8%), neck stiffness (7/9; 77.8%), and photophobia (3/9; 33.3%). Seizures occurred in one patient (1/9; 11.1%). No patient had an altered level of consciousness. Neuroimaging was normal in 7/9 patients (77.8%); two patients (2/9; 22.2%) had abnormal findings. Eight of nine patients (8/9; 88.9%) achieved full neurological recovery. Total hospital stay ranged from 2 to 15 days (median: 3 days); PICU stay ranged from 1 to 12 days (median: 1 day). Demographic and clinical features are summarized in Table 1, and laboratory parameters, management, and outcomes in Table 2.

**Table 1:** Demographic and clinical characteristics (n = 9). Data are presented as N (%) for categorical variables. The shaded bottom row shows the total count and percentage of patients with each presenting feature. As this is a descriptive case series (n = 9), formal inferential statistical testing was not performed. Statistical significance threshold for any future comparative analyses: p < 0.05.

Case	Age / Sex	Fever	Headache	Vomiting	Photophobia	Neck Stiffness	Seizures
1	8 yr / F	Yes	Yes	Yes	Yes	Yes	No
2	8 yr / M	Yes	Yes	Yes	Yes	Yes	No
3	6 yr / M	Yes	Yes	Yes	Yes	No	No
4	6 wk / M	Yes	No	No	No	No	No
5	10 yr / M	Yes	Yes	Yes	No	Yes	No
6	17 mo / M	Yes	No	Yes	No	Yes	Yes
7	5 yr / M	Yes	Yes	Yes	No	Yes	No
8	5 yr / M	Yes	Yes	Yes	No	Yes	No
9	5 yr / F	Yes	Yes	Yes	No	Yes	No
Total	9 patients	9/9 (100%)	8/9 (88.9%)	8/9 (88.9%)	3/9 (33.3%)	7/9 (77.8%)	1/9 (11.1%)

**Table 2:** Laboratory parameters, management, and clinical outcomes. Data are presented as individual patient values with a summary row showing the median (continuous variables) and N/% (categorical variables). ᵃPCT reported for 8/9 patients (88.9%); Case 8 value not measured. Pediatric reference ranges: Blood WBC: 4.5–11.0 ×10⁹/L; CRP: < 10 mg/L; PCT: < 0.5 ng/mL; CSF WBC: 0–5/mm³; CSF protein: 0.15–0.45 g/L; CSF Glucose: 2.2–4.4 mmol/L. WBC: white blood cell count; CRP: C-reactive protein; PCT: procalcitonin; CSF: cerebrospinal fluid; ALERD: acute leukoencephalopathy with restricted diffusion; WM: white matter; Abx: antibiotic therapy; PICU: pediatric intensive care unit; N/A: not available.

Case	Blood WBC (×10⁹/L)	CRP (mg/L)	PCT (ng/mL)	CSF WBC (/mm³)	CSF Protein (g/L)	CSF Glucose (mmol/L)	Neuroimaging	Abx (days)	PICU (days)	Total Stay (days)	Outcome at Discharge
1	6.1	37.5	0.28	5	0.50	3.35	Normal	2	2	3	Full recovery
2	8.57	19.1	0.42	55	0.22	3.36	Normal	1	1	2	Full recovery
3	15.0	21.0	0.15	86	0.31	3.42	Normal	1	1	2	Full recovery
4	9.78	29.7	0.11	5	0.69	2.86	Not performed	1	1	2	Full recovery
5	9.8	60.0	0.20	30	0.26	5.33	Cerebral edema + meningeal enhancement	2	2	4	Full recovery
6	9.3	3.1	25.0	2	0.17	5.80	ALERD (bilateral frontal + parietal WM)	6	12	15	Persistent deficits (epilepsy, motor)
7	9.5	25.0	0.25	550	0.40	3.19	Normal	3	2	4	Full recovery
8	9.1	17.1	N/Aᵃ	1	0.16	3.60	Normal	1	1	4	Full recovery
9	9.7	4.7	0.10	75	0.40	2.90	Normal	1	1	3	Full recovery
Median	9.5	21.0	0.27ᵃ	30	0.31	3.36	7/9 normal (77.8%)	1	1 [1–2]	3 [2–4]	8/9 full recovery (88.9%)

Individual case descriptions

Case 1

An eight-year-old female presented with fever, headache, vomiting, photophobia, and neck stiffness, without seizures. Laboratory evaluation showed a blood WBC count of 6.1 × 10⁹/L, CRP of 37.5 mg/L, and PCT of 0.28 ng/mL. CSF analysis revealed 5 WBCs/mm³, protein 0.50 g/L, and glucose 3.35 mmol/L, while CSF PCR confirmed enterovirus infection. Neuroimaging findings were normal. The patient received empirical antibiotics for two days and had a total hospital stay of three days, including two days in the PICU. She made a full recovery and remained asymptomatic at the 2-4-week follow-up.

Case 2

An eight-year-old male presented with fever, headache, vomiting, photophobia, and neck stiffness, without seizures. Laboratory investigations showed a blood WBC count of 8.57 × 10⁹/L, CRP of 19.1 mg/L, and PCT of 0.42 ng/mL. CSF analysis revealed 55 WBCs/mm³, protein 0.22 g/L, and glucose 3.36 mmol/L, while CSF PCR was positive for enterovirus. Neuroimaging findings were normal. The patient received empirical antibiotics for one day and had a total hospital stay of two days, including one day in the PICU. He recovered completely, with no sequelae noted at follow-up.

Case 3

A six-year-old male presented with fever, headache, vomiting, and photophobia, without neck stiffness or seizures. Laboratory investigations showed a blood WBC count of 15.0 × 10⁹/L, CRP of 21.0 mg/L, and PCT of 0.15 ng/mL. CSF analysis revealed 86 WBCs/mm³, protein 0.31 g/L, and glucose 3.42 mmol/L, while CSF PCR confirmed enterovirus infection. Neuroimaging findings were normal. The patient received empirical antibiotics for one day and had a total hospital stay of two days, including one day in the PICU. He recovered completely and remained asymptomatic at follow-up.

Case 4

A six-week-old male infant presented with fever alone, without headache, vomiting, photophobia, neck stiffness, or seizures. Laboratory investigations showed a blood WBC count of 9.78 × 10⁹/L, CRP of 29.7 mg/L, and PCT of 0.11 ng/mL. CSF analysis revealed 5 WBCs/mm³, protein 0.69 g/L, and glucose 2.86 mmol/L, while CSF PCR confirmed enterovirus infection. Neuroimaging was not performed, as the clinical presentation was mild and imaging was not indicated in this six-week-old infant. The patient received empirical antibiotics for one day and had a total hospital stay of two days, including one day in the PICU. He recovered fully, with no complications noted at follow-up.

Case 5

A 10-year-old male presented with fever, headache, vomiting, and neck stiffness, without photophobia or seizures. Laboratory investigations showed a blood WBC count of 9.8 × 10⁹/L, CRP of 60.0 mg/L, and PCT of 0.20 ng/mL. CSF analysis revealed 30 WBCs/mm³, protein 0.26 g/L, and glucose 5.33 mmol/L, while CSF PCR was positive for enterovirus. Brain MRI demonstrated cerebral edema with meningeal enhancement. The patient received empirical antibiotics for two days and had a total hospital stay of four days, including two days in the PICU. He made a full recovery and remained asymptomatic at follow-up.

Case 6

**Figure 1:**
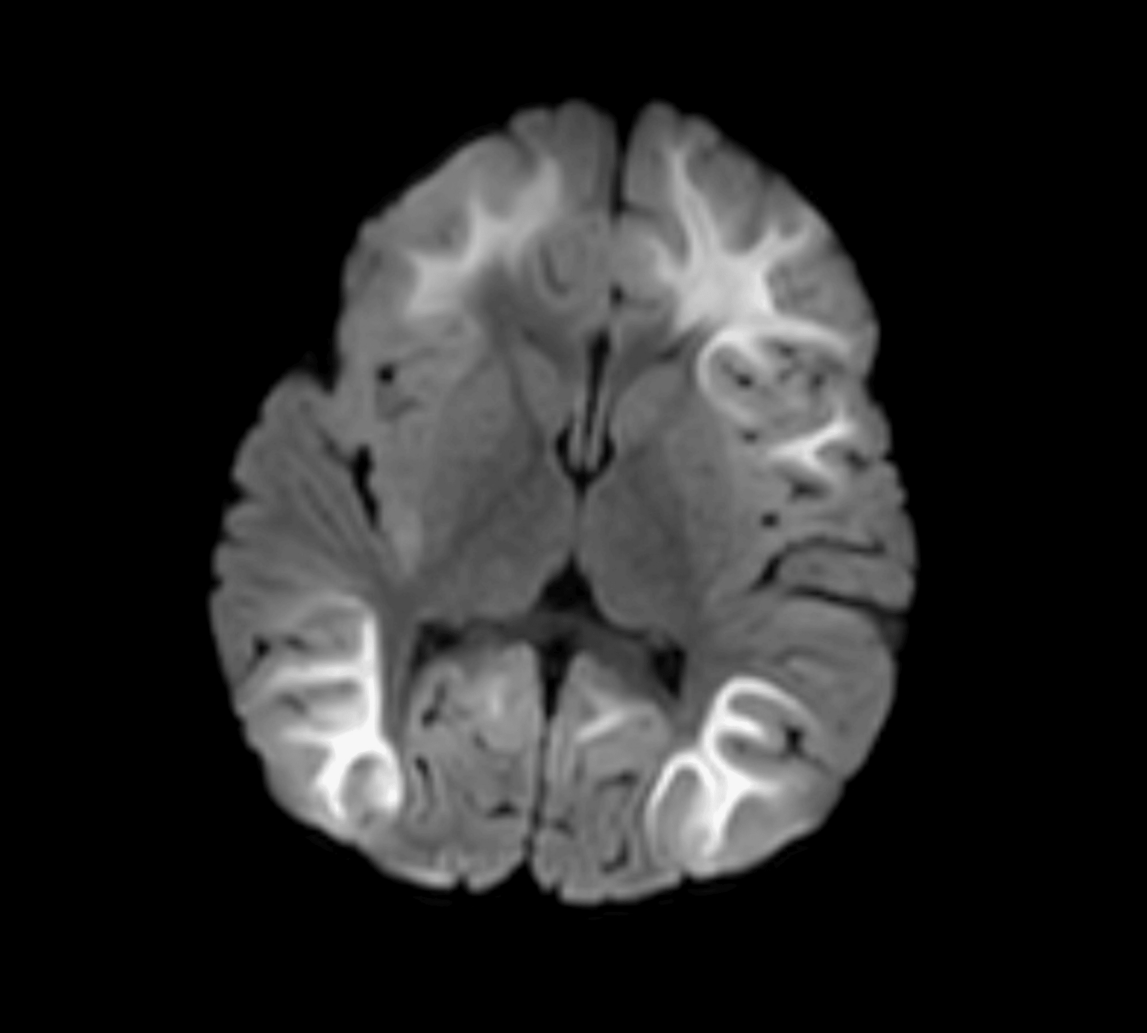
Representative brain MRI — diffusion-weighted imaging (DWI) demonstrating ALERD in case 6 (patient 6: 17-month-old male with enteroviral encephalitis). Axial DWI brain MRI from Case 6 (17-month-old male) demonstrating extensive bilateral symmetric areas of bright diffusion restriction (high signal on DWI) involving the subcortical and periventricular white matter of the bilateral posterior frontal and parietal lobes, with additional involvement of the occipital white matter. These findings are consistent with acute leukoencephalopathy with restricted diffusion (ALERD), a rare but distinct pattern of brain injury associated with severe enteroviral encephalitis. In the appropriate clinical context, acute viral illness, CSF PCR-confirmed enterovirus, and rapid neurological deterioration, these bilateral symmetric white matter diffusion abnormalities are characteristic of ALERD and carry prognostic significance for adverse neurological outcomes. Sequence: DWI b=1000. The patient was 17 months of age at the time of imaging.

A 17-month-old male presented with fever, vomiting, neck stiffness, and seizures. Laboratory investigations showed a blood WBC count of 9.3 × 10⁹/L, CRP of 3.1 mg/L, and markedly elevated PCT of 25.0 ng/mL. CSF analysis revealed 2 WBCs/mm³, protein 0.17 g/L, and glucose 5.80 mmol/L, while CSF PCR confirmed enterovirus infection. Brain MRI with diffusion-weighted imaging (DWI) demonstrated extensive bilateral symmetric diffusion restriction involving the subcortical and periventricular white matter of the frontal and parietal lobes, internal and external capsules, and patchy regions of the corpus callosum, findings consistent with acute leukoencephalopathy with restricted diffusion (ALERD) (Figure 1). The patient required mechanical ventilation for three days and received empirical antibiotics for six days, corticosteroids, and intensive supportive care. Acyclovir was administered for one day and discontinued once CSF PCR confirmed viral etiology. Hospital stay was 15 days (12 in PICU). At discharge: impaired vocalization, loss of sitting balance, lower limb spasticity, inability to stand independently, and new-onset epilepsy. At the 12-week follow-up: no active seizures on tapering levetiracetam, regained independent ambulation.

Case 7

A five-year-old male presented with fever, headache, vomiting, and neck stiffness, without photophobia or seizures. Laboratory investigations showed a blood WBC count of 9.5 × 10⁹/L, CRP of 25.0 mg/L, and PCT of 0.25 ng/mL. CSF analysis demonstrated marked pleocytosis with 550 WBCs/mm³, protein 0.40 g/L, and glucose 3.19 mmol/L, while CSF PCR confirmed enterovirus infection. Neuroimaging showed no evidence of cerebral edema or meningeal enhancement. The patient received empirical antibiotics for three days and had a total hospital stay of four days, including two days in the PICU. He recovered fully, with no neurological sequelae observed at follow-up.

Case 8

A five-year-old male presented with fever, headache, vomiting, and neck stiffness, without photophobia or seizures. Laboratory investigations showed a blood WBC count of 9.1 × 10⁹/L and CRP of 17.1 mg/L; procalcitonin was not measured. CSF analysis revealed 1 WBC/mm³, protein 0.16 g/L, and glucose 3.60 mmol/L, while CSF PCR confirmed enterovirus infection. Neuroimaging findings were normal. The patient received empirical antibiotics for one day and had a total hospital stay of four days, including one day in the PICU. He made a full recovery and remained asymptomatic at follow-up.

Case 9

A five-year-old female presented with fever, headache, vomiting, and neck stiffness, without photophobia or seizures. Laboratory investigations showed a blood WBC count of 9.7 × 10⁹/L, CRP of 4.7 mg/L, and PCT of 0.10 ng/mL. CSF analysis revealed 75 WBCs/mm³, protein 0.40 g/L, and glucose 2.90 mmol/L, while CSF PCR was positive for enterovirus. Neuroimaging showed no evidence of cerebral edema or meningeal enhancement. The patient received empirical antibiotics for one day and had a total hospital stay of three days, including one day in the PICU. She recovered completely, with no neurological deficits noted at follow-up.

Correlation analyses

**Figure 2:**
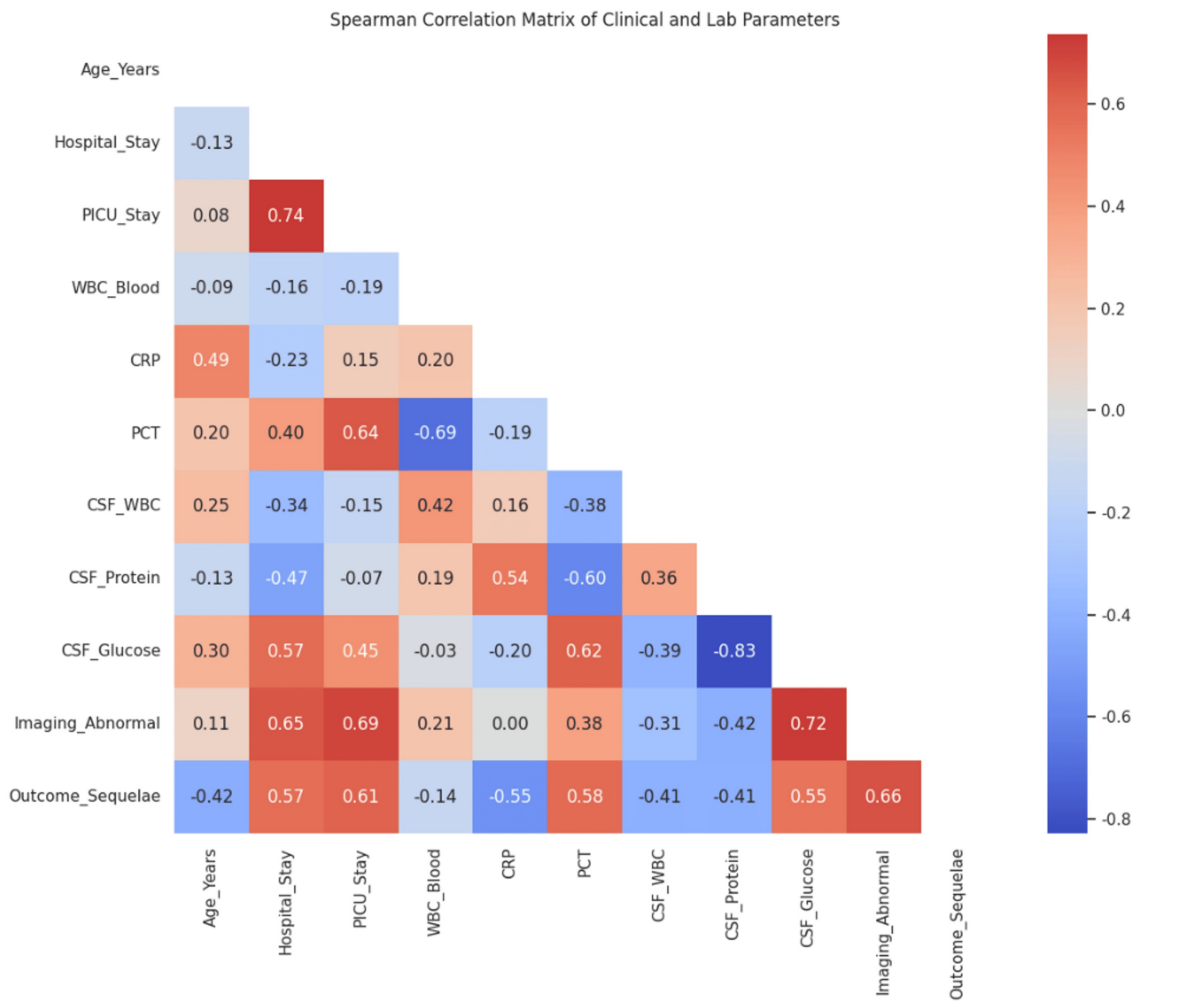
The Spearman correlation matrix of clinical and laboratory parameters Spearman rank correlation matrix of clinical and laboratory parameters (n = 9). Each cell displays the Spearman correlation coefficient (rs). Statistical significance was defined as p < 0.05 (two-tailed). A significant positive correlation was identified between abnormal neuroimaging (cerebral edema or enhancement) and PICU stay duration (rs = 0.694, p = 0.038). CRP did not significantly correlate with clinical outcome (rs = −0.548, p = 0.127). CRP: C-reactive protein; PCT: procalcitonin; CSF WBC: cerebrospinal fluid white blood cell count; PICU: pediatric intensive care unit.

The following analyses are exploratory and hypothesis-generating only and should not be interpreted as confirmatory evidence, given the small sample size of nine patients. Spearman’s rank correlation analysis demonstrated an association between abnormal neuroimaging and prolonged PICU stay (rs = 0.694, p = 0.038). CRP did not corelate with clinical outcome (rs =− 0.548, p = 0.127). The patient with the highest CRP (60.0 mg/L) achieved full recovery, while the patient with the most severe outcome (Case 6) had a markedly elevated PCT (25.0 ng/mL) despite a low CRP. The full correlation matrix is presented in Figure 2. The inherent limitations of Spearman correlation in a cohort of nine patients, including susceptibility to individual outliers, absence of multiple comparisons correction, and low statistical power, must be kept in mind when interpreting these results.

## Discussion

This retrospective descriptive case series documents nine children admitted with PCR-positive enteroviral encephalitis at Burjeel Hospital, Abu Dhabi, over a 12-month period. Several observations merit discussion: the predominance of meningeal over encephalitic features at presentation, the generally favorable clinical course, the exploratory association between neuroimaging abnormalities and clinical severity, and the profile of one patient who sustained severe and evolving neurological injury.

Fever was universal (9/9; 100%), with vomiting (8/9; 88.9%), headache (7/9; 77.8%), and neck stiffness (7/9; 77.8%) occurring in the majority. Seizures were documented in only one child (1/9; 11.1%), and no patient had an altered level of consciousness, a distribution more consistent with aseptic meningitis than with classical encephalitis. Classical teaching associates encephalitis with a higher incidence of seizure activity and altered consciousness [2,7]. The low seizure rate in our series likely reflects the predominantly meningitic phenotype of most cases rather than true encephalitic parenchymal involvement. This is consistent with the mild to moderate CSF pleocytosis and largely normal neuroimaging across the cohort. We acknowledge that some patients may be better classified as aseptic meningitis with mild encephalitic features, and this diagnostic nuance should be considered when interpreting these findings.

CSF findings were consistent with the expected profile of viral CNS infection: lymphocytic pleocytosis of varying degrees, near-normal glucose levels, and mildly elevated protein in most patients. Importantly, the degree of CSF pleocytosis did not correspond with clinical severity or outcome. Patient 7, who had the highest CSF white cell count (550 cells/µL), had an uncomplicated course and full recovery. This is well recognized in enteroviral infection and reflects that CSF cellularity is a marker of the host inflammatory response rather than an index of parenchymal injury [3,5]. CSF pleocytosis alone should not be used to escalate therapy or predict prognosis.

CSF PCR was positive for enterovirus in all nine patients (100%) and was central to clinical decision-making, enabling timely de-escalation of empirical antimicrobial therapy within 24-48 hours of admission in all cases. We used a pan-enteroviral PCR platform; enterovirus serotyping was not performed, which limits subtype-specific analysis [8].

In this exploratory series, neuroimaging findings were the clinical feature most consistently associated with adverse outcomes. Seven patients (7/9; 77.8%) had normal brain imaging, and all achieved full neurological recovery. The two patients (2/9; 22.2%) with imaging abnormalities followed divergent courses: one had cerebral edema with meningeal enhancement and fully recovered after a prolonged PICU admission; the other (Case 6) had MRI features of ALERD, with extensive bilateral diffusion restriction involving the frontal and parietal white matter, internal capsules, and corpus callosum (Figure 1). ALERD is a rare but distinct entity associated with severe outcomes in enteroviral encephalitis [9]. The exploratory Spearman correlation between abnormal neuroimaging and prolonged PICU stay (rs = 0.694, p = 0.038) cannot be regarded as confirmatory given the sample size but aligns with the established clinical principle that early MRI, particularly DWI sequences, is warranted in children with severe or deteriorating enteroviral CNS infection.

Systemic inflammatory markers, specifically CRP and white cell count, did not predict clinical outcome. The patient with the highest CRP and leukocytosis made a rapid and complete recovery. Case 6, who sustained the most severe neurological injury, had markedly elevated PCT (25.0 ng/mL). Whether this reflected bacterial co-infection, systemic inflammatory spillover from fulminant brain injury, or an independent process is difficult to determine retrospectively. The absence of a correlation between peripheral inflammatory markers and neurological outcome is consistent with evidence that severe enteroviral encephalitis may be driven by direct viral cytopathic effects and neuroinflammatory mechanisms not captured by systemic markers [10].

Management was primarily supportive across the cohort: careful fluid balance, antipyretics, and anticonvulsants were used when indicated. Empirical antibiotics were initiated at admission in all patients and de-escalated within 24-48 hours once viral etiology was established. No steroids were routinely used. Acyclovir was administered to Case 6 for one day and discontinued once CSF PCR confirmed enteroviral etiology, an approach consistent with good antimicrobial stewardship.

One patient (1/9; 11.1%) stands out as a critical clinical outlier. Case 6 presented with seizures, had ALERD on MRI within 24 hours (Figure 1), and had the highest PCT in the cohort. He required a 12-day PICU stay and was discharged with significant neurological deficits. At the 12-week follow-up, meaningful recovery was documented: no active seizures on tapering levetiracetam and regained independent ambulation, but persistent deficits remained. The co-occurrence of seizures at presentation, ALERD on neuroimaging, and markedly elevated PCT in a single patient is a hypothesis-generating observation only; it cannot be regarded as a validated risk-stratification tool on the basis of this series alone and warrants prospective evaluation in larger cohorts.

The epidemiological profile is consistent with the known biology of enteroviral infection. The age range of six weeks to ten years reflects the recognized susceptibility of young children lacking prior immunity [11]. Male predominance (7/9; 77.8%) has been observed in some but not all published series and may reflect reporting bias in small cohorts as much as genuine biological difference. The clinical and virological patterns observed in our cohort appear broadly consistent with international reports, but local enteroviral serotypes, environmental factors, or host genetic determinants may modulate disease expression in ways not detectable from this small series.

Limitations of this study

The limitations of this study must be clearly acknowledged. First, the retrospective single-center design and small sample size (n = 9) substantially restrict statistical power and limit generalizability; no conclusion from this series can be applied to broader populations without prospective validation. Second, the exploratory Spearman correlation (rs = 0.694, p = 0.038) is not confirmatory, is vulnerable to type I error in a small cohort, and should be interpreted with considerable caution. Third, the distinction between true encephalitis and aseptic meningitis was not always definitively established; some patients may represent predominantly meningitic involvement. Fourth, enterovirus serotyping was not performed, precluding serotype-specific outcome analysis. Fifth, no correction for multiple comparisons was applied. Sixth, the 4-12-week follow-up is insufficient to characterize long-term neurodevelopmental outcomes. Future research should include multicenter prospective cohort studies with standardized diagnostic criteria, serotyping, and long-term follow-up.

## Conclusions

This is a descriptive, hypothesis-generating case series of nine children with PCR-confirmed enteroviral encephalitis. The findings should be interpreted within the limitations described above and do not constitute confirmatory evidence. In this cohort, the illness predominantly followed a mild clinical course: all nine patients survived, 8/9 (88.9%) achieved full neurological recovery, and hospital stays were brief (median: three days). However, one patient (1/9; 11.1%) with seizures at presentation and ALERD on DWI-MRI (Figure 1) sustained severe neurological injury, illustrating that the clinical spectrum of enteroviral encephalitis can can occasionally result in severe injuries. In this exploratory series, abnormal neuroimaging was the clinical feature most consistently associated with prolonged PICU stay and adverse outcome; this warrants prospective evaluation in larger cohorts. Early CSF PCR testing enabled timely antibiotic de-escalation in all cases, consistent with good antimicrobial stewardship. These findings contribute region-specific descriptive data from the UAE and support the importance of early neuroimaging, particularly DWI-MRI, and systematic neurodevelopmental follow-up in children with enteroviral CNS infection. Prospective multicenter studies with standardized criteria, enterovirus serotyping, and long-term follow-up are required to validate these observations.
